# Mesenchymal Stem Cell-Derived Exosomes: Biological Function and Their Therapeutic Potential in Radiation Damage

**DOI:** 10.3390/cells10010042

**Published:** 2020-12-30

**Authors:** Xiaoyu Pu, Siyang Ma, Yan Gao, Tiankai Xu, Pengyu Chang, Lihua Dong

**Affiliations:** 1 Jilin Provincial Key Laboratory of Radiation Oncology & Therapy, Department of Radiation Oncology & Therapy, The First Bethune Hospital of Jilin University, Changchun 130021, China; puxy19@mails.jlu.edu.cn (X.P.); masy19@mails.jlu.edu.cn (S.M.); gao_yan@jlu.edu.cn (Y.G.); xutk16@mails.jlu.edu.cn (T.X.); 2 Key Laboratory of Organ Regeneration & Transplantation of the Ministry of Education, Department of Radiation Oncology & Therapy, The First Bethune Hospital of Jilin University, Changchun 130021, China; 3 National Health Commission Key Laboratory of Radiobiology, School of Public Health, Jilin University, Changchun 130021, China

**Keywords:** mesenchymal stem cell, exosome, radiation damage

## Abstract

Radiation-induced damage is a common occurrence in cancer patients who undergo radiotherapy. In this setting, radiation-induced damage can be refractory because the regeneration responses of injured tissues or organs are not well stimulated. Mesenchymal stem cells have become ideal candidates for managing radiation-induced damage. Moreover, accumulating evidence suggests that exosomes derived from mesenchymal stem cells have a similar effect on repairing tissue damage mainly because these exosomes carry various bioactive substances, such as miRNAs, proteins and lipids, which can affect immunomodulation, angiogenesis, and cell survival and proliferation. Although the mechanisms by which mesenchymal stem cell-derived exosomes repair radiation damage have not been fully elucidated, we intend to translate their biological features into a radiation damage model and aim to provide new insight into the management of radiation damage.

## 1. Introduction

Mesenchymal stem cells (MSCs) are multipotent stem cells that can be isolated from human tissues or organs, such as the bone marrow, adipose tissue, umbilical cord, lung, spleen, liver or kidney [1]. Despite being derived from multiple sources, MSCs display similar biological phenotypes and functions [2,3]. Because of their autocrine and paracrine actions, MSCs have been shown to possess potency in repairing tissue damage [4]. Critically, delivery of only a small population of MSCs can result in accelerated damage repair in the host [5,6,7]. In addition, exosomes are crucial components that account for the paracrine action of MSCs [8,9,10]. For example, they exchange genetic material across cells by transferring bioactive molecules [11]. Similar to other cellular exosomes, MSC-exosomes are extracellular vesicles with a lipid bilayer structure and an average diameter of 100 nm [1,12]. They carry bioactive molecules, including miRNAs, lncRNAs, lipids and cytokines [1], thus providing a context for researching the biological functions of MSC-exosomes.

Treating diseases with MSC-exosomes has shown promise in the field of regenerative medicine, and numerous studies exploring the therapeutic effects of MSC-exosomes on neurological, immunological and cardiovascular diseases have been published [13]. In summary, the benefits of delivering MSC-exosomes in disease models mainly include the attenuation of inflammation, promotion of angiogenesis and improvement in the survival and proliferation of stem or progenitor cells within injured tissues or organs [14]. In fact, such benefits can be achieved with MSCs as well. Although it has also been shown that MSCs can exert therapeutic effects on radiation damage, the therapeutic potential of MSC-exosomes has not been widely explored in this field. Nevertheless, in a previous study, irradiated cells exhibited enhanced uptake of exosomes because of an increase in the formation of the integrin and tetraspanin complex CD29/CD81 on the cell surface [15], thus indicating the specific role of exosomes in mediating biological processes in injured cells. Moreover, MSC-exosomes were found to protect against acute or chronic radiation damage via their miRNA cargo, suggesting that irradiated cells might utilize MSC-exosomes to increase their resistance to ionizing irradiation [16,17,18]. For example, a study showed that exosomal miRNA-210 could elicit efficient DNA damage repair by controlling the transcriptional activity of HIF-1, thus enhancing cellular radio-resistance [17,19]. In this review, we explore the pro-regenerative properties of MSC-exosomes in the field of radiation damage and aim to provide new insight into the management of radiation damage by using MSC-exosomes.

## 2. Biological Features of MSC-Exosomes

MSCs are crucial sources of exosomes in humans. Consistent with other cell-derived exosomes, MSC-exosomes are generated through a sequential process including the invagination of lysosomal microparticles and fusion and excretion from parental cells [20]. Lysosomal microparticles first invaginate their membranes to generate endosomes, which then fuse with each other to form multivesicular bodies that contain intraluminal vesicles. Next, the outer membrane of the mature multivesicular body fuses with the plasma membrane of a cell and is ultimately transported out, constituting an exosome [20].

Exosomes consist of lipid bilayer membrane structures with diameters ranging from 40 nm to 160 nm (an average of 100 nm) [12]. They express various markers, including CD9, CD81, CD63, TSG101, flotillin, ceramide, and Alix [12], and have a density of 1.15–1.19 g/mL in sucrose gradients [21]. MSC-exosomes contain at least 170 different miRNAs [22] and 304 proteins [23], along with an indefinite number of DNAs, mRNAs and metabolites [12]. Because they contain a large number of bioactive molecules, MSC-exosomes have attracted great interest in the field of regenerative medicine. Accordingly, numerous studies have attempted to assess whether the infusion of MSC-exosomes can serve as an alternative strategy to repair tissue damage, and emerging results have mostly revealed that MSC-exosomes have therapeutic effects similar to those of their parental MSCs [24]. Moreover, MSC-exosomes have several advantages over MSCs. (i) MSC-exosomes are long-lasting and can be stored at −80 °C without affecting their biological functions [17], whereas cryopreserved MSCs exhibit impaired immunoregulatory and pro-regenerative properties compared with fresh MSCs [25]. (ii) The membranes of MSC-exosomes are enriched in sphingomyelin, cholesterol, ceramide and lipid raft proteins, enabling MSC-exosomes to spread in vivo regardless of biological barriers, such as the blood-brain barrier [26], for example, even when they are delivered via an intravenous injection, MSC-exosomes can be detected in injured neurons in the brain [27]. (iii) Infusion of MSC-exosomes elicits minimal immune rejection due to their complete lack of expression of major histocompatibility complex (MHC) molecules [28,29], which prevents their rapid clearance by host immune cells. For instance, MSC-exosomes were found to remain in a recipient for a significantly longer time than MSCs after infusion [28,30], indicating that they can perform their biological functions in vivo for a relatively long time. (iv) Infusion of MSC-exosomes can avoid several stem cell-associated challenges, such as the risk of spontaneous tumorigenesis induced by MSCs [31,32]. (v) The potential secretion of exosomes by MSCs can be impacted by various factors. For example, maintaining MSCs in a physiological state in an in vitro culture system can impact their production of exosomes with a specific phenotype in terms of biological activity [33]. Notably, although incubating MSCs with an IFN-γ plus TNF-α mixture in vitro reduced their proliferation, the production of exosomes was not adversely affected [28,34]. Moreover, this process improved the immunosuppressive function of the MSC-exosomes. This prompts speculation that exosomes with high bioactivity can be purposefully obtained by preconditioning MSCs in vitro prior to injection to treat inflammatory diseases. Therefore, determining the components of MSC-exosomes that are able to produce high therapeutic efficacy is particularly critical.

The miRNA and protein cargo contained in MSC-exosomes are effective in promoting damage repair. Moreover, they jointly regulate the regenerative process in damaged tissue. In a colitis model, MSC-exosomes were revealed to reduce macrophage-induced inflammation by transporting metallothionein-2, an upstream protein that blocks activation of the NF-κB pathway [28]. However, this anti-inflammatory effect of MSC-exosomes was not completely lost even when blocking metallothionein-2 in vivo and in vitro [28], demonstrating that other components in MSC-exosomes also exert bioactive effects in this process. Therefore, exosomal miRNA-146a in MSCs might alleviate experimental colitis by targeting the *TRAF6* and *IRAK1* genes [35], preventing NF-κB activation along with the subsequent production of TNF-α and IL-6 [35]. Consistently, several other MSC-exosomal miRNAs such as miRNA-30b-3p [36], miRNA-223-3p [37], and miRNA-126 [38,39] were found to be responsible for suppressing pro-inflammatory responses. They also exhibit potent effects in promoting tissue regeneration and angiogenesis. Overall, we need to understand the mechanisms by which MSC-exosomes repair tissue damage.

## 3. Therapeutic Functions of MSC-Exosomes

### 3.1. Immunomodulation

To our knowledge, commoditized MSCs have been approved for treating some autoimmune diseases in a clinical setting; however, the incidence of infection secondary to infusion of allogenic MSCs has been reported to be 29.5% [40]. This has prompted us to find an alternative approach. Exosomes are thought to be superior over MSCs with regard to treatment-related safety [41]. A previous work suggested that MSC-exosomes improved the in vitro survival and function of neutrophils from patients with severe congenital neutropenia, thus increasing the potential efficacy of MSC-exosomes against acute infection [42]. Moreover, studies have suggested that MSC-exosomes exhibit effects in managing autoimmune or inflammatory diseases [37,43] (Table 1).

In vitro, MSC-exosomes exert an immunomodulatory function, mainly by regulating the commitment of immune cells or altering their inflammatory cytokine secretion profiles [28]. For example, in the presence of IFN-γ and TNF-α, MSCs generate exosomes that induce macrophages to switch from an M1- to an M2-like phenotype, and exosomal miRNAs, including miRNA-146 and miRNA-34, greatly contribute to this process [34]. Mechanistically, miRNA-146 upregulate expression of M2-associated genes such as *TRAF6* and *IRAK1* by targeting NF-κB signaling [53], and miRNA-34 targets *Notch1* to suppress transcription of genes encoding M1-related pro-inflammatory cytokines, such as IL-6 and TNF-α [54]. The MSC-exosomal miRNA-181a has been consistently found to enhance the production of M2-related cytokines, including IL-10 and TGF-β while reducing production of the M1-related cytokines TNF-α, IL-6 and IL-12 by macrophages [46,55]. In addition to altering the secretion profile of macrophages, miRNA-181a induces Treg cell generation by suppressing expression of the *c-Fos* gene, which functionally counteracts the Foxp3-dominant transcriptional program associated with Treg cell development [45]. Nevertheless, a coculture experiment revealed that Treg cell induction by MSC-exosomes was less efficient than that by MSCs, indicating that some other factors contribute to this process. In fact, soluble factors from MSCs, including IDO, PGE2 and IL-10 strongly induce Treg cell generation [56]; however, except for IL-10, they are not present in MSC-exosomes [28,50].

**Figure 1:**
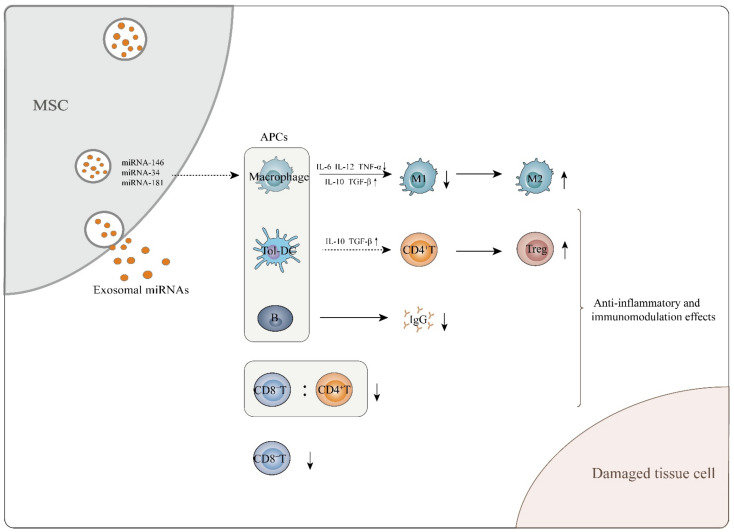
MSC-exosomes exert anti-inflammatory and immunomodulatory effects, which aid in attenuating tissue cell damage. MSC-exosomes perform these functions mainly through interaction of exosomal miRNAs and APCs. They can induce immature and mature DCs to differentiate into tolerogenic DCs, mediating naïve CD4^+^ T cell differentiation into Tregs. In addition, MSC-exosomes are able to induce macrophages to transform from the M1 to the M2 phenotype while enhancing secretion of M2-related cytokines such as IL-10 and TGF-β and decreasing M1-related cytokine TNF-α, IL-6 and IL-12 levels. With regard to B cells, MSC-exosomes inhibit the maturation and function of B lymphocytes and cause a decrease in IgG secretion. MSC-exosomes also decrease the CD8^+^ T cell number and the CD8^+^/CD4^+^ T cell ratio in the peripheral blood of in vivo mouse models.

In vivo, MSC-exosomes control immunomodulatory processes in an antigen-presenting cell (APC)-mediated manner [52]. For example, dendritic cells (DCs) serve as critical mediators of the effects of MSC-exosomes on Treg induction. Mechanistically, MSC-exosomes induce mature DCs to acquire immune tolerogenic phenotypes [51]. A critical function of tolerogenic DCs is inducing Treg cell generation in vivo [57]. Tolerogenic DCs secrete high levels of anti-inflammatory cytokines, such as IL-10 and TGF-β, and express low levels of costimulatory molecules, thus inducing naïve CD4^+^ T cells to commit to differentiation into Tregs [51]. In addition to DCs, MSC-exosomes are able to restrict B cell maturation, which decreases the production of immunoglobulin-G (IgG) [50,58]. To a certain extent, the above effects of MSC-exosomes will assist in attenuating the immune responses driven by other T subsets, such as Th1, Th2, Th17 cells or CD8^+^ T cells [33,59,60,61] (Figure 1). Indeed, studies have shown that incubating mouse adipose tissue-derived MSC-exosomes with mouse splenic immunocytes in vitro significantly downregulates expression of genes encoding Tbx21, Gata3 and Rorc, which centrally control the commitment of Th1, Th2 and Th17 cells, respectively [47]. CD8^+^ T cells that delivered human umbilical cord-derived MSC-exosomes to GVDH mice significantly decreased the number of CD8^+^ T cells along with the ratio of CD8^+^ T cells to CD4^+^ T cells in the peripheral blood [59]. However, intriguingly, reduced numbers of CD4^+^ or CD8^+^ T cells did not occur when conditioned by MSC-exosomes in vitro, which suggests, at least, that MSC-exosomes modulate host immune responses independently of their direct effect on impairing the survival of CD4^+^ or CD8^+^ T cells [50] (Figure 1). In other words, the mechanisms by which MSC-exosomes induce immunomodulation in vivo are more complicated than those observed in vitro, and the details need to be further elucidated.

### 3.2. Angiogenesis

Angiogenesis has a crucial role in tissue regeneration after damage. In this process, endothelial cells invade injured tissues to form buds and ultimately establish a capillary network. It is well accepted that MSCs exert therapeutic effects on ischemic diseases by directly producing or stimulating endogenous factors, such as vascular endothelial growth factor (VEGF), hepatocyte growth factor (HGF) and stromal-derived factor-1 (SDF-1) [62,63]. These factors facilitate angiogenesis in damaged tissues [64]. Recent evidence has revealed that MSC-exosomes also have pro-angiogenic properties [60,65] (Table 2).

**Figure 2:**
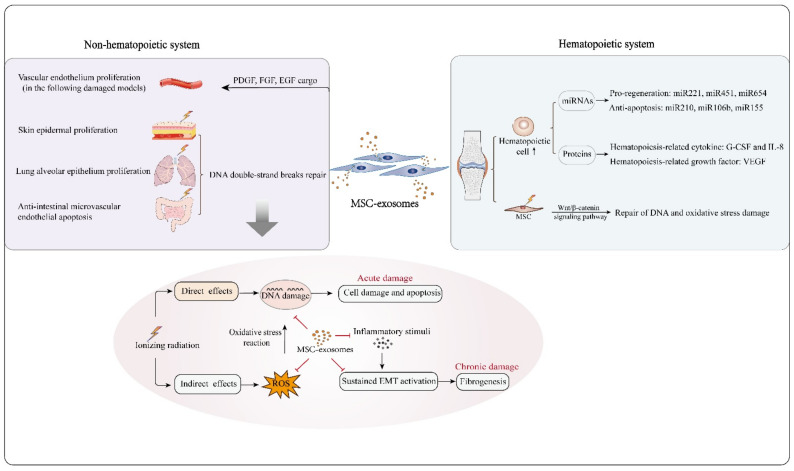
MSC-exosomes are capable of protecting against radiation-induced damage to hematopoietic and nonhematopoietic systems. In hematopoietic reconstruction post irradiation, MSC-exosomes enhance hematopoietic cell survival and proliferation by carrying functional molecules, such as the pro-regeneration miRNAs miRNA221, miRNA451 and miRNA654, the anti-apoptosis-related miRNAs miRNA210, miRNA106b and miRNA155, the hematopoiesis-related cytokines G-CSF and IL-8, and the hematopoiesis-related growth factor VEGF. In addition, MSC-exosomes can protect irradiated bone marrow MSCs from radiation-induced DNA and oxidative stress damage by activating the Wnt/β-catenin signaling pathway. With regard to the non-hematopoietic system, MSC-exosomes reduce apoptosis of skin epidermal, lung alveolar epithelium and intestinal epithelium cells, as MSC-exosomal miRNAs likely mediate repair of DNA double-strand breaks in damaged cells. Oxidative stress reaction and DNA damage are the major processes in radiation damage. MSC-exosomes can overcome these crucial events effectively, and have potential to suppress the development of acute and chronic radiation damage from several aspects. MSC-exosomes also facilitate vascular endothelium proliferation owing to their bioactive cargo molecules, such as PDGF, FGF and EGF.

Based on proteomic analysis, MSC-exosomes contain various factors that are involved in angiogenesis, such as platelet-derived growth factor (PDGF), fibroblast growth factor (FGF), epidermal growth factor (EGF) and proteins associated with NF-κB activation [61]. PDGF, FGF and EGF function as common factors in mediating angiogenesis [77], and the role of proteins associated with NF-κB activation in mediating angiogenesis should be addressed. To our knowledge, NF-κB activation is conventionally associated with inducing pro-inflammatory responses. Nonetheless, intriguingly, blocking NF-κB activation abrogated tube formation by endothelial cells in vitro [61]. Consistent with this finding, exosomes from bone marrow MSCs were found to activate STAT3 signaling cascades in target cells, thus upregulating expression of genes encoding HGF, insulin-like growth factor-1 (IGF1), nerve growth factor (NGF) and SDF-1 [63]. Similarly, several other studies have revealed the mechanisms by which MSC-exosomes induce angiogenesis. For example, exosomes from umbilical cord MSCs reportedly activate Wnt/β-catenin to increase angiogenesis [75], but those from bone marrow MSCs promote angiogenesis by activating the HIF-1α/VEGF axis in target cells [73]. Furthermore, bone marrow MSCs increase the survival of pulmonary endothelial cells via exosomal miRNA-21-5p, which targets the antioncogenes *PDCD4* and *PTEN* in a mouse model of ischemia/reperfusion [78]. In addition, placental MSC-exosomes are capable of upregulating expression of genes encoding Ang2 and Tie2 by endothelial cells [60]. The details of the angiogenic features of MSC-exosomes are provided in Table 2 and Figure 2.

Similar to their immunomodulatory features, the pro-angiogenic potency of MSC-exosomes can be impacted by foreign stimuli [55,69]. For example, it was found that MSCs conditioned with PDGF showed increased production of exosomes containing angiogenic molecules, such as c-kit and stem cell factor [55]. However, preconditioning MSCs with pro-inflammatory cytokines, such as TNF-α and IL-6, increased the exosomal cargo content of miRNA-196a-5p and miRNA-17-5p, inactivating the PI3K-AKT, MAPK and VEGF-related pathways and impairing angiogenesis [79]. In addition to such bioactive substances, environmental factors impact the proangiogenic properties of MSC-exosomes. In fact, exposure of MSCs to blue light resulted in the increased content of miRNA-135b-5p and miRNA-499a-3p as exosomal cargo, which promoted angiogenesis in vitro by repressing myocyte enhancer factor 2C (MEF2C) [66]. Consistent with this finding, hypoxia enhanced the cargo content of miRNA-126 in MSC-exosomes. In this context, miRNA-126 was able to stimulate SPRED1/Ras/Erk/HIF-1α, thus increasing angiogenesis in injured tissues [38]. More intriguingly, HIF-1α was found to upregulate expression of the gene encoding RAB22A, which participates in vesicle formation in cells [80]. This event partially illustrates why HIF-1α-overexpressing MSCs can increase their production of exosomes [38]. Functionally, exosomes from cells such MSCs promote angiogenesis by activating the Jagged-1/Notch signaling pathway [55]. As the pro-angiogenic potency of MSC-exosomes can be improved by using the above methods, we can purposely generate them and utilize them to treat ischemic diseases (Table 2).

### 3.3. Epithelial Recovery

Apart from their effects on immunomodulation and angiogenesis, a growing body of evidence has revealed the therapeutic effects of MSC-exosomes on epithelial injuries. In summary, MSC-exosomes increase the proliferation and survival of epithelial cells.

MSC-exosomes accelerate epithelial recovery in wounded tissues via their miRNA cargo. By using different disease models, recent studies have reported some specific roles of MSC-exosomal miRNAs in mediating epithelial recovery, such as that of miRNA-135a in increasing epithelial cell migration by suppressing expression of the gene encoding LATS2 during cutaneous wound healing [81] and that of miRNA-126 in activating the PI3K-AKT and MAPK pathways during cutaneous healing in a rat model of diabetes [82]. Moreover, exosomes may carry specific cargo such as foreign miRNA products. For example, in a study of MSCs genetically modified to overexpress a variety of miRNAs, including miRNA-100, miRNA-146a, miRNA-21, miRNA221 and miRNA-143, it was found that these exosomes enhance DNA synthesis, thus promoting the proliferation of vaginal epithelial cells [83]. In an acute lung injury model, exosomes from miRNA-30b-3p-overexpressing MSCs protected type II alveolar epithelial cells against apoptosis by downregulating serum amyloid A3 (SAA3) [36].

In fact, increasing cell proliferation and survival by activating PI3K/Akt and MAPK are typical effects of both MSCs and their exosomes. Other effects of MSC-exosomes during epithelial recovery should be mentioned, including antioxidation. To our knowledge, oxidation is a harmful occurrence that impairs cell survival. In a renal injury model, MSC-exosomes inhibited apoptosis in tubular epithelial cells by reducing the level of reactive oxygen species (ROS) [84]. As documented, mitochondrial dysfunction is an important biological event that is closely associated with lung disease pathogenesis and/or progression [85]. Mechanistically, MSC-exosomes improve the mitochondrial function of lung epithelial cells by targeting division/fusion-related genes such as *rhot1*, *mfn1* and *opa1* [86]. Simultaneously, exosomes have been shown to carry functional mitochondria and promote mitochondrial transfer events [87], further demonstrating that MSC-exosomes have the potential to alleviate mitochondrial damage and control the progression of tissue damage.

MSC-exosomes also inhibit the epithelial-mesenchymal transition (EMT) [88,89,90], which is critical in inducing tissue fibrosis, resulting in pathological rather than functional restoration of damaged tissue. Although the underlying mechanisms by which MSC-exosomes restrict fibrotic development are not clear, MSC-exosomes inhibit activation of the TGF-β1/Smad pathway [91] while enhancing expression of zona occludens protein-1 in epithelial cells, which is related to cellular tight junctions [92]. Therefore, MSC-exosomes at least reduce epithelial depletion due to transformation, thereby maintaining the integrity of the epithelium and suppressing tissue fibrosis. Collectively, MSC-exosomes promote epithelial recovery by facilitating regeneration, inhibiting apoptosis and reducing EMT depletion.

## 4. Role of MSC-Exosomes in Repairing Radiation Damage

Despite the use of advanced treatment techniques, radiation damage is common and often unavoidable in cancer patients during or after receiving radiotherapy. The actions of ionizing radiation on biological molecules can be segmented into direct and indirect effects. DNA damage in cells can be induced by the direct effects of ionizing radiation, and it can also be caused by the oxidative stress reaction mediated by reactive oxygen species (ROS) generated by indirect effects. Ionizing irradiation-induced DNA double-strand breaks, oxidative stress, vascular damage, and subsequent inflammation are typical events in the acute phase of the pathogenesis of radiation damage, and if these events are not well managed, fibrosis occurs as a pathogenic feature in the chronic phase [55].The potential use of MSCs in repairing radiation-induced acute damage in the hematopoietic system, liver, lung, gastrointestinal tract, or skin has been explored [88,93,94,95], and the results indicate that MSCs have several therapeutic features including increased proliferation and survival of tissue/organ-specific stem/progenitor cells, the promotion of angiogenesis, anti-inflammation and oxidation, and the reduction of fibrotic pathogenesis [96]. The above findings indicate that MSC-exosomes have similar potencies to those of MSCs in repairing tissue or organ damage due to disease. Moreover, recent advances have demonstrated the repair of radiation damage by MSC-exosomes. In the following sections, we elaborate on the therapeutic effects of MSC-exosomes on radiation damage in the hematopoietic system and nonhematopoietic system (Figure 2).

Hematopoietic cells are sensitive to radiation exposure, which can lead to bone marrow failure. Several studies have shown that MSC-exosomes are capable of repairing radiation-induced hematopoietic system injury, but the exact mechanism is unclear. A few studies have suggested that the following processes may contribute to the relevant mechanism. (i) MSC-exosomes can transfer miRNAs with pro-regenerative or anti-apoptotic effects to irradiated hematopoietic cells. For example, intravenous delivery of human bone marrow MSC-derived extracellular vesicles (MSC-EVs, mainly comprising exosomes and microvesicles) swiftly normalized the counts of peripheral blood cells in mice that received whole-body irradiation because their cargo content, including miRNA-221, miRNA-451 and miRNA-654-3p, promoted the proliferation of irradiated marrow cells and miRNA210-5p, miRNA106b-3p and miRNA155-5p prevented radiation-induced hematopoietic cell apoptosis [17]. (ii) MSC-exosomes can restore hematopoiesis by stimulating secretion of hematopoiesis-related cytokines. A previous study has suggested that human placental MSCs rescue radiation-induced hematopoiesis in mice by secreting human hematopoiesis-related cytokines, including G-CSF, MCP-1, IL-6 and IL-8 [16], and this effect can be observed with MSC-exosomes as well [18]. In fact, the data from a recent study show that MSC-exosomes are capable of inducing production of high levels of hematopoiesis-related cytokines such as G-CSF, IL-6, IL-8 and VEGF by macrophages in vitro [18]. (iii) MSC-exosomes have several other features that cause the remodeling of hematopoietic cells. For example, incubation with MSC-exosomes enhances the activity of macrophages, which are regarded as the key regulators of demand-adapted hematopoiesis [89]. MSC-exosomes are also able to directly restore irradiated bone marrow MSCs, which are considered to be potent contributors to hematopoiesis. One critical mechanisms involves the alleviation of DNA damage and oxidative stress via Wnt/β-catenin signaling pathway activation [90].

In addition to remodeling the hematopoietic system, MSC-exosomes are capable of protecting the skin, gastrointestinal system, respiratory system and other systems against radiation damage. At the micro level, radiation damage is essentially attributed to the large number of oxygen free radicals generated by ionizing radiation, which subsequently result in DNA double-strand breaks. Previous studies have reported that MSCs play a key role in alleviating DNA damage and oxidative stress damage [97]. MSC-exosomes, the functional role of which depend on their cargo derived from cells of origin, exert similar remodeling effects [98,99,100]. For example, in an oxidative stress-induced skin injury model, MSC-exosome treatment decreased ROS generation and subsequent DNA damage and improved the antioxidant capacities of damaged cells through NRF2 signaling [100]. Other studies have found that after MSC-exosome treatment in an ischemic renal disease model, damaged renal cells showed reduced oxidative stress marker (MDA) levels, increased anti-oxidant marker (SOD and CAT) levels, and significantly reduced DNA damage parameters [98]. However, the underlying molecular mechanism is poorly understood. Notably, studies have shown that some miRNAs that are contained in MSC-exosomes such as miRNA210 are able to repair DNA double-strand breaks [17,19], which suggests that exosomes may exert remodeling functions in a noncoding RNA-mediated epigenetic manner. This may explain the decrease in the apoptosis of skin epidermal cells, lung alveolar epithelium, intestinal epithelium and various parenchymal cells after MSC-exosome exposure [60,101,102,103]. Such noncoding RNA cargo may affect nonhomologous end-joining (NHEJ), which is common and essential in mammalian cell DSB repair [104]. Current evidence suggests that MSC-exosomes treatment is beneficial for the repair of oxidative stress-induced damage [22,105,106], although the exact functional components remain to be revealed. In addition, it has been found that intravenously injected MSC-EVs (including exosomes) are highly distributed in parenchymal organs such as the liver and spleen in a whole-body irradiation mouse model [105]. This may provide the context for the development of cures for radiation-induced parenchymal organ injury. Microvascular endothelial apoptosis has been recognized as the primary process that initiates radiation-induced injury [106]. Studies have found that local MSC-exosome treatment can facilitate the proliferation of vascular endothelial cells by activating the Wnt4/β-catenin pathway [75,103]. MSC-exosomes also have the potential to cure radiation-induced injury partly due to their potent pro-angiogenic factor cargo such as PDGF, FGF and EGF [61], which induce endothelial proliferation and differentiation in vitro and neovascularization in vivo [107,108].

On the other hand, exosomal targeting of cells is mediated by members of the integrin and tetraspanin families or other associated molecules based on their expression [109,110]. An experimental study demonstrated that radiation contributes to increased formation of the integrin and tetraspanin complex CD29/CD81 on the cell surface, thus enhancing uptake of exosomes by irradiated cells [15]. This further illustrates the potential use of MSC-exosomes in the treatment of radiation damage. In general, the repair effect of MSC-exosomes on radiation damage in multiple systems is partly attributed to their bioactive cargo, which predominately consists of noncoding RNAs and functional proteins. These molecules influence the expression of target genes associated with radiation-induced damage or tissue regeneration due to epigenetic regulation. Overall, more experimental studies are required to further explore the molecular mechanisms involved.

## 5. MSC-Exosomes in Repairing Radiation Damage: Perspective and Challenges

With regard to radiation-induced damage, it has been revealed that MSCs play a crucial role in tissue damage treatment and prevention. Moreover, the superior properties and improved safety of MSC-exosomes make them novel candidates for curing radiation-induced damage. They exert therapeutic effects mainly by facilitating angiogenesis, promoting cellular regeneration, and probably by enhancing the repair function through immunomodulatory effects. More importantly, there are several methods that can be used to enhance the efficacy of remodeling damaged tissue. On the one hand, exosomes secreted by MSCs with genetic modifications are a promising alternative treatment, such as exosomes derived from SDF1-overexpressing MSCs for microvascular regeneration [111]. On the other hand, MSCs can be pretreated in vitro before exosomes are collected, such as with hypoxia-treated MSC-exosomes in ischemia-related disease [55]. Last, but equally important, the tropism of exosomes can be improved by increasing expression of specific receptors on the surface of the original MSCs. Current studies on the treatment of radiation-induced damage by MSC-exosomes are mostly based on the acute phase, whereas little work has been performed on the treatment of chronic radiation-induced damage by MSC-exosomes. Notably, evidence suggests that MSC-exosomes reverse EMT of endometrial epithelial cells via the TGF-β1/Smad pathway [91] and of tubular epithelial cells via enhanced tight junctions [92]. In general, sustained EMT is a critical mechanism that underlies the fibrotic pathology of tissue [112]. Thus, it can be reasonably inferred that MSC-exosome treatment has potential for preventing tissue fibrosis in the chronic phase of tissue damage. Therefore, despite limited evidence of the repair role of MSC-exosomes in chronic radiation-induced damage, it is important that researchers make further efforts to explore their therapeutic and underlying potential in chronic radiation-induced damage. This will provide a new context for the future application of MSC-exosomes to treating chronic radiation damage (Figure 2).

In fact, there are several deficiencies with regard to managing diseases by using MSC-exosomes. (i) One concern is the challenges due to the instability of contents of exosomes. For example, studies have shown that the amount of exosomal miRNA cargo is influenced by the irradiation dose and pH value of the culture medium [113,114]. The precise experimental conditions for exosomes are more difficult to control compared to MSCs. (ii) Another concern is the lack of a uniform standard for the purification and quantification of exosomes from conditioned media. Overall, it is difficult to determine the equivalent dose of exosomes in dose-dependent experimental studies, which may lead to different conclusions as results can be affected by exosome content and impurities. Therefore, it is appropriate to find an ideal method for constructing a precise equivalent dose of exosomes for experimental purpose. Although the effects of MSC-exosomes in various disease models have been clearly shown, the exact components and mechanisms of therapy are not entirely clear. miRNAs and functional proteins may play major roles, yet the role of MSC-exosomes in tumor growth and metastasis remains controversial. Previous studies have shown that MSC-exosomes can promote tumor growth in vivo [115], but a recent study revealed that MSC-exosomes enhance radiotherapy-induced tumor cell death in primary and metastatic tumor foci through synergistic and bystander effects [116]. Urgent issues for cancer patients receiving radiotherapy include the adjuvant antitumor effect and resistance to radiation damage. There is a great need for researchers to elaborate on the role of MSC-exosomes in regenerative medicine for the treatment of radiation damage.

## 6. Conclusions

MSC-exosomes show potential for repairing radiation damage. Current data reveal that MSC-exosomes have therapeutic potential due to their anti-inflammatory effects and promotion of angiogenesis and epithelial survival, which are crucial biological processes in the remodeling of radiation damage. In addition, the immunomodulatory effects of MSC-exosomes probably enhance their tissue repair function. Overall, MSC-exosomes have good prospects for the treatment of radiation injury and this may inspire future research in this field.

## Figures and Tables

**Table 1:** Mesenchymal Stem Cell (MSC) Exosomes Perform Immunoregulation Effects.

Models In Vivo/Vitro	Exosome Source	Immunoregulation Effects	Ref.
**In Vivo Models**			
Inflammatory bowel disease mice	Human bone marrow MSCs	Increased levels of anti-inflammatory cytokines IL-10; Decreased levels of pro-inflammatory IL-1β, IL-6, IFN-γ and TNF-α; Promotion of the M2b macrophage polarization.	[28]
Inflammatory bowel disease mice	Human Umbilical Cord MSCs	Increased levels of IL-10; Decreased levels of IL-1β, IL-6, TNF-α, iNOS, and IL-7; Inhibition of macrophages infiltration into the colon tissues.	[43]
Inflammatory bowel disease mice	Human Umbilical Cord MSCs	Increased levels of IL-10 and IP-10; Decreased levels of TNF-α, IL-1β, IL-6.	[44]
Myocardial ischemia/reperfusion mice	Human Umbilical Cord MSCs	Increased levels of anti-inflammatory cytokine IL-10 and decreased levels of proinflammatory cytokines TNF-α and IL-6 via exosomal miRNA-181a; Promotion of Treg cell development.	[45]
Autoimmune encephalomyelitis rats	Rat bone marrow MSCs	Increased M2-related anti-inflammatory cytokines of IL-10 and TGF-β; Decreased M1-related proinflammatory TNF-α and IL-12 levels; Promotion of the M2 phenotype microglia polarization.	[46]
Autoimmune encephalomyelitis mice	Mice adipose MSCs	Decreased Tbx21 and Gata3 expression, as the crucial regulator for Th1 and Th2 cell responses; Decreased Rorc and Elf4 expression, as the activator and inhibitor for Th17 differentiation.	[47]
Acute lung injury mice	Rat bone marrow MSCs	Decreased expression of pulmonary TNF-α, IL-1β and IL-6 via inhibiting TLR4-NF-κB signaling pathway.	[48]
Neuroinflammation rats	Rat bone marrow MSCs	Reduced oxidative stress responses; Decreased levels of IL-1β, IL-6 and TNF-α; Inhibition of neuronal degeneration and apoptosis.	[49]
Autoimmune Hepatitis mice	Mice bone marrow MSCs	Attenuation of liver inflammation via carrying miRNA-223-3p; Increased Treg/Th17 ratio; Decreased levels of IL-1β and IL-6.	[37]
Inflammatory arthritis mice	Mice bone marrow MSCs	Decreased CD8^+^T cell frequency and CD8^+^/CD4^+^ T cell ratio in vivo; No reduction in CD8^+^T cell and CD8^+^/CD4^+^ T cell ratio in vitro; Promotion of Treg populations.	[50]
**In Vitro Co-Culture Models of Immunocytes with Exosomes**			
Bone marrow-derived dendritic cells (BMDCs)	Mice adipose MSCs	Inhibition of the BMDCs proliferation; Transformation of immature and mature DCs into tolerogenic DCs	[51]
Macrophages	Human adipose MSCs	Shift of macrophages from M1 to M2 phenotype polarization via shuttling functional miRNA-146.	[34]
Peripheral blood mononuclear cells (PBMCs)	Human bone marrow MSCs	Increased secretion of IL-10 and TGF-β1 from PBMCs; Promotion of the Treg cells differentiation.	[52]
PBMCs	Human bone marrow MSCs	Inhibition of the B lymphocytes proliferation and differentiation; Decreased levels of immunoglobulin secretion.	[47]
Neutrophils	Human adipose MSCs	Augment ROS production of neutrophils; Promotion of lifespan and function of neutrophils.	[42]

**Table 2:** MSC Exosomes Facilitate Angiogenesis in Various Disorders.

Models	Exosome Source	MSCs Disposure	Carry	Angiogenic Mechanisms	Ref.
**In Vivo Models**					
Severe combined immunodeficiency mice	Human adipose MSCs	PDGF-stimulated	c-kit with its ligand SCF	Increasing matrix metalloproteinases content and enhancing the angiogenic potential via c-kit/SCF.	[55]
Hind limb ischemia mice	Human placental MSCs	NO-stimulated	miR-126	Promoting angiogenesis by increasing VEGF and miR-126 levels.	[39]
Cutaneous burn mice	Human umbilical cord MSCs	Blue light-stimulated	miR-135b-5p; miR-499a-3p	Promoting angiogenic activity via the upregulation of functional miR-135b-5p; miR-499a-3p.	[66]
Athymic-nude mice	Human dental pulp MSCs	HIF-1-overexpressed	Jagged 1	Enhancing Jagged 1-mediated angiogenesis through Notch signaling pathway	[67]
Bone fracture mice	Human umbilical cord MSCs	Hypoxia- treated	miR-126	Promoting angiogenesis and bone fracture healing through HIF-1α/miR-126 and SPRED1/Ras/Erk signaling pathways.	[38]
Nude mice	Human adipose MSCs	Hypoxia- treated	-	Promoting angiogenesis by increasing the expression of VEGF and activating the PKA signaling pathway.	[68]
Nude mice	Human adipose MSCs	Hypoxia- treated	-	Promoting angiogenesis at least partially through upregulating VEGF/VEGF-R signaling pathway.	[69]
Calvarial defect rats	Human bone marrow MSCs	DMOG-stimulated	-	Promoting angiogenesis by activation of the AKT/mTOR signaling pathway	[70]
Acute myocardial infarction rats	Human umbilical cord MSCs	Akt-transfected	-	Accelerating angiogenesis via upregulating PDGF-D expression.	[71]
Immunodeficient mice	Human adipose MSCs	-	miRNA-125a	Promoting angiogenesis by transferring miR-125a to endothelial cells and repressing angiogenic inhibitor delta-like 4.	[65]
Femoral head osteonecrosis mice	Human bone marrow MSCs	-	miRNA-224-3p	Promoting angiogenesis by downregulating exosomal microRNA-224-3p.	[72]
Femora fracture rats	Human bone marrow MSCs	-	-	Promoting angiogenesis and osteogenesis via activation of the HIF-1α/VEGF and the BMP-2/Smad1/RUNX2 signaling pathways.	[73]
Femoral fracture rats	Human umbilical cord MSCs	-	-	Promoting angiogenesis and fracture healing through increasing the expression of VEGF and HIF-1α.	[74]
Cutaneous wound rats	Human umbilical cord MSCs	-	-	Promoting angiogenesis via activating the Wnt4/β-catenin signaling pathway in a dose-dependent manner.	[75]
Auricle ischemic injury mice	Human placental MSCs	-	-	Stimulating angiogenic activity in endothelial cells via upregulating their responsiveness to proangiogenic growth factors.	[60]
Acute myocardial infarction rats	Rat bone marrow MSCs	-	-	Enhancing the density of new functional capillary and promoting blood flow recovery; Inhibiting proliferation and function of T cells; Reducing infarct size and preserving cardiac systolic and diastolic performance	[64]
**In Vitro Co-Culture of Human Cells with Exosomes**					
Human umbilical vein endothelial cells and fibroblasts	Human bone marrow MSCs	-	Transcriptionfactor STAT3	Inducing proliferation and migration of fibroblasts; Promoting the expression of growth factors, like HGF, IGF1, NGF and SDF1; Activating Akt, Erk, and STAT3 signaling pathways, which are both involved in angiogenesis.	[63]
Human umbilical vein endothelial cells and fibroblasts	Human bone marrow MSCs	-	Wnt3a protein	Enhancing fibroblast proliferation, migration; Promoting angiogenesis via carrying Wnt3a protein in vitro.	[76]
